# Covid-19 Pandemic: What Changes for Dentists and Oral Medicine Experts? A Narrative Review and Novel Approaches to Infection Containment

**DOI:** 10.3390/ijerph17113793

**Published:** 2020-05-27

**Authors:** Maria Eleonora Bizzoca, Giuseppina Campisi, Lorenzo Lo Muzio

**Affiliations:** 1 Department of Clinical and Experimental Medicine, University of Foggia, 71121 Foggia, Italy; marielebizzoca@gmail.com; 2 Department of Surgical, Oncological and Oral Sciences (Di.Chir.On.S.), University of Palermo, 90121 Palermo, Italy; campisi@odonto.unipa.it; 3 C.I.N.B.O. (Consorzio Interuniversitario Nazionale per la Bio-Oncologia), 66100 Chieti, Italy

**Keywords:** COVID-19, SARS-CoV-2, personal protective equipment (PPE), dentistry, oral medicine

## Abstract

The authors performed a narrative review on Severe Acute Respiratory Syndrome- CoronaVirus-2 ( SARS-CoV-2) and all infectious agents with the primary endpoints to illustrate the most accepted models of safety protocols in dentistry and oral medicine, and to propose an easy view of the problem and a comparison (pre- vs post-COVID19) for the most common dental procedures. The outcome is forecast to help dentists to individuate for a given procedure the differences in terms of safety protocols to avoid infectious contagion (by SARS-CoV-2 and others dangerous agents). An investigation was performed on the online databases Pubmed and Scopus using a combination of free words and Medical Subject Headings (MESH) terms: “dentist” OR “oral health” AND “COVID-19” OR “SARS-CoV-2” OR “coronavirus-19”. After a brief excursus on all infectious agents transmittable at the dental chair, the authors described all the personal protective equipment (PPE) actually on the market and their indications, and on the basis of the literature, they compared (before and after COVID-19 onset) the correct safety procedures for each dental practice studied, underlining the danger of underestimating, in general, dental cross-infections. The authors have highlighted the importance of knowing exactly the risk of infections in the dental practice, and to modulate correctly the use of PPE, in order to invest adequate financial resources and to avoid exposing both the dental team and patients to preventable risks.

## 1. Introduction

The era of Corona-Virus-Disease-19 (COVID-19) is an important historical period from various points of view, from the world health to the huge cascade of socio-economic implications. Everyday habits have been turned upside down, and the way of life of people all over the globe, engaged in all activities, especially in the health sector, will be involved in this necessary change. Dentists, being in close contact with the patient’s droplets and aerosols generated, have to revise the operating protocols to protect the team and the patients from the risk of infectious diseases.

Unfortunately, the pandemic COVID-19 will not stop immediately and everyone will have to face each other very long working and social recovery times of the population. In this time, a large part of the population will avoid dental treatment other than those imposed by pain or urgency, both due to money issues and, principally, for a psychological reason: it will not be easy to overcome the fear of infection. For many, the dental practice is a source of possible infections, considering that the first person at risk is the dentist himself. The scenario in dental practices is very complex and several problems can arise which are dangerous for the dental practice [1,2,3,4,5,6,7,8,9].

For an infection to emerge, it is necessary that an adequate number of specific microorganisms can infect a person or groups. The classic contamination paths clearly incorporate all the dental unit (team and patient): body fluids in direct contact with the wound site during operation, injuries of the skin and the mucosa with sharp objects, body fluids and contaminated material contact with eyes, aerosols arising during the operation with air produced by turbine and ultrasonic devices, contamination via droplet, and surgical smoke formed during electro-cautery or laser applications [10,11]. 

The first problem raised with respect to COVID-19, is related to the easy spread of viral agents in the air during dental procedures [12,13,14,15]. Hence, aerosol is the most aggressive source of COVID-19 as well as other viral infections, placing dentists and their collaborators at the first line of the exposure to risk scale within the context of healthy personnel [10,16].

The second problem is related to the persistence of the biological agent in operating rooms. The aerosol produced by high rotation instruments and ultrasound could remain for several hours in the air and on the surfaces [17,18]. Although it can save the operator, if well protected, during the therapeutic acts, it means that the air will be contaminated, thus presenting a risk for operators after removing the PPE (personal protective equipment) and for the next patients.

This COVID-19 pandemic has shown that several people can be positive and spread the viral agents around without any symptoms or signs of biological agents. So, the dental team, a part performing the double triage [1], should consider each patient as SARS-COV-2 positive until proven otherwise and use protective equipment in order to preserve their own health and the health of all patients as attending the dental office.

For these reasons, it is necessary to use rigid and precise operating protocols capable of classifying dental procedures based on risks for the team as well as for the patients.

This study was born from the awareness of a necessary change in decision making processes. It involves a rereading of relevant literature in order to build protocols addressed to dentists, to assess and modulate the risks of contagion in the dental practice. Moreover, it proposes, on the basis of information from literature, a classification of dental procedures based on the risk of contagion of infectious agents, showing what will change for the dentist and the oral medicine expert.

## 2. Materials and Methods 

**Figure 1:**
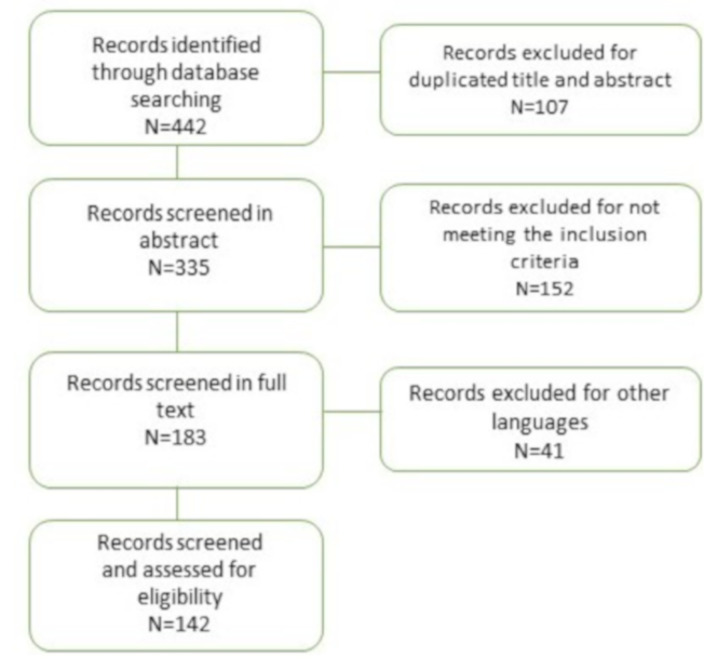
flow chart showing articles selection process.

An investigation was performed on the online databases Pubmed and Scopus using a combination of free words and MESH terms: “dentist” AND “COVID-19” OR “SARS-CoV-2” OR “coronavirus-19”, and “oral health” AND “COVID-19” OR “SARS-CoV-2” OR “coronavirus-19”. Only studies fulfilling the following inclusion criteria were considered eligible for inclusion in this study: (i) performed on human subjects, (ii) written in the English language, and (iii) published in 2019–2020. The manuscript titles list was highlighted to exclude irrelevant publications and search errors. The final selection was performed by reading the full texts of the papers in order to approve each study’s eligibility based on SARS-COV-2 and other infective agents involved in dentistry. Data selection and revision was performed by two independent reviewers (MEB, University of Foggia and GC, University of Palermo). They singularly analysed the papers, and in agreement, included 142 papers in this narrative review. The authors, in consideration of the importance of the emerging topic, decided to include also guidelines, online documents, reviews, experts’ opinions, renouncing the PRISMA-related design of regular systematic reviews (Figure 1).

## 3. Results

After a brief excursus on all possible infectious agents, the authors described, on the basis of the literature selected, all personal protective equipment (PPE) actually on the market and their indications. Then, they compared (before vs after COVID-19 era) the correct safety procedures for each dental practice selected, underlining the danger of underestimating, in general, dental cross infections, if focused only on the newest SARS-CoV-2. Results are summarised in Tables 1–8.

### 3.1. Infectious Agents

#### 3.1.1. Transmission Mode in Healthcare Settings

**Figure 2:**
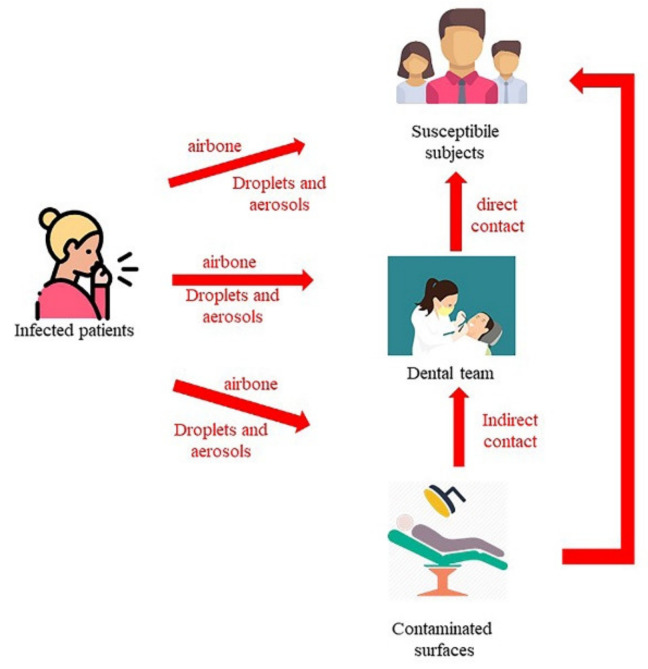
the three main routes of transmission for infectious agents.

Different classes of bacteria, viruses, and fungi can cause human infections. Three factors are important for the transmission of these infectious agents: an infectious agent, a receptive subject and a transmission mode. The pathogens involved in infections during health care mainly derive from staff, from patients (and possible careers), but also from inanimate environmental sources (Figure 2).

These human sources can: 1) have active infections, 2) be asymptomatic or in an incubation period, or 3) be colonized transiently or chronically with pathogen microorganisms.

The infection is the consequence of the contact between a contagious agent and a potential host. Moreover, the same characteristics of the host can influence the onset and the severity of the infectious disease. However, several other factors can modify the virulence and behavior of infectious disease such as the number of infectious agents, the transmission way and the pathogenicity [19]. Predictors of the disease evolution in a specific subject could be: immune status at exposition time, age, comorbidity, and virulence of the agent [20].

There are two main ways of infective transmission, namely vertical (from mother to fetus: transplacental, during vaginal birth or breast feeding) and horizontal (sexual and non-sexual). In a dental setting, infectious agents are transmitted in the horizontal, non-sexual route [21].

In the non-sexual horizontal transmission, direct or indirect contact (e.g., Herpes simplex virus, respiratory syncytial virus, *S. aureus*), droplets (e.g., influenza virus, B. pertussis) or airways (e.g., *M. tuberculosis*) are possible routes. Other viruses can be transmitted by the blood (e.g., Hepatitis B and C viruses and HIV) via percutaneous or mucous membrane exposure [3,4,14,22]. In synthesis, the three main routes of the transmission are [23]:

- Contact transmission: Contact transmission can be through direct contact and indirect contact.
  ○ During direct contact transmission, pathogens are transmitted from an infected person to another subject without an intermediate object or person (for example, mucous membrane or breaks contact blood or other blood-containing body fluids infected, or contact HSV lesion without gloves) [3,4,14,15,18,22,24,25].
  ○ During indirect contact transmission, pathogens are transmitted to the host through objects or human body carrying those pathogens [17,18,22,26,27,28,29,30]. Moreover, all the personal protective equipment (PPE), such as uniforms or isolation gowns, can be contaminated by infectious agents during the treatment of a patient colonized or infected.
- Droplet transmission: Some infectious agents can reach the host through the direct and indirect contact routes or through droplets [3,15,31,32,33]. Droplets can carry infectious pathogens travelling for short distances directly from the respiratory tract of the infectious subjects to host reaching susceptible mucosal surfaces [3,15,31,32,33]. Respiratory droplets are produced during coughs, sneezes, or talks [34] or by airway health procedures. The nasal mucosa, conjunctivae, and mouth are good portals for respiratory viruses [35]. To date, the maximum distance that a droplet can reach is not known, even if pathogens transmitted by a droplet do not run across long distances [19]. The size of droplets has traditionally been defined as being >5 µm [19]. Several types of droplets, including those with diameters of 30 µm or greater, can remain suspended in the air [36]. The sizes of the droplets can determine the maximum distance reached: largest droplets, between 60 and 100 microns, totally evaporate before spontaneously falling 2 m away [37]. For respiratory exhalation flows, the critical factor is the exhalation air velocity: these droplets are carried more than 6 m away by exhaled air at a velocity of 50 m/s (sneezing), more than 2 m away at a velocity of 10 m/s (coughing), and less than 1 m away at a velocity of 1 m/s (breathing) [37].
- Airborne transmission: This means of transmission consists of dissemination of airborne droplet or small particles containing infectious pathogens that remain infective over time and distance (e.g., spores of *Aspergillus* spp., and *M. tuberculosis*) [31,33,38,39,40,41,42].

#### 3.1.2. Infectious Agents of Particular Importance in Dentistry Settings 

Several infectious agents can involve dentist and his team [10] (Table 1).

##### Viral infections

- SARS-COV-2 determines COVID-19 (coronavirus disease 2019), an infectious disease characterized by several important systemic problems such as coronavirus associated pneumonia. The principal symptoms are fever, cough, and breathing difficulties; the most patients have mild symptoms, some progress to severe pneumonia [43]. The diagnosis is performed with the identification of the virus in swabs of patient throat and nose. COVID-19 can involve the respiratory tract determining a mild or highly acute respiratory syndrome due to the production of pro-inflammatory cytokines, such as interleukin (IL)-1beta and IL-6 [44]. One mechanism that can make the coronavirus lethal is the induction of interstitial pneumonia linked to an over-production of IL-6 [44,45]. Based on this principle, several researchers have started to use an anti-arthritis drug, tocilizumab, for its anti-IL-6 action [46,47,48,49].
- Herpes simplex virus (HSV) can determine a primary infection with minor, ulcerative lymphadenopathy gingivostomatitis [50] and a recurrent infection with cold sores. Herpetic whitlow, an HSV infection of the fingers is usually caused by direct contact of the same fingers with infected saliva or a herpetic lesion [51,52,53]. Skin, mucosal lesions, and secretions such as saliva can determine the transmission [54,55]. Lesions are usually characterized by vesicles and sequent crusting. Acyclovir can be used for the treatment of the diseases. It is sufficient to wear gloves in order to avoid the herpetic whitlow when the clinician treats patients with HSV lesions [10,56].
- Varicella zoster virus (VZV) can determine chickenpox (primary disease), usually in children, and shingles, which is very painful (secondary disease), for the reactivation of a virus residing in sensory ganglia during the latency period [57,58,59]. Chickenpox disease is highly contagious and spreads via-airborne routes [60,61,62]. The virus can infect nonimmune dental team via inhalation of aerosols from a patient incubating the disease. Masks and gloves can be not sufficient for complete protection of the healthcare workers [10].
- Epstein–Barr virus can determine mononucleosis, while epithelial tissues can be the site of latency for this virus. Skin contact, blood, or saliva can transmit the virus, thus members of the dental team are considered to be in the risk group of EBV infection [63,64,65,66,67,68].
- Human herpes virus 6 (HHV6) can determine generalized rash. The virus is present in saliva and a dental team can be considered at low-risk [69,70].
- Influenza, rhino- and adenoviruses, are respiratory viruses. For this reason, they can be transmitted by droplets and dental team is at risk. However, masks and gloves can adequately protect healthcare workers [10,71,72,73].
- Rubella (German measles) is a toga virus that can cause cataract, deafness, and other complications which affect developing foetus, so it is particularly dangerous for the female components of a dental team during pregnancy. It can be transmitted by droplets. In order to avoid these problems, dental staff could be vaccinated for MMR (measles, mumps, and rubella) [10,74,75,76].
- Coxsackie virus causes hand-foot and mouth disease and herpangina [77]. It is present in saliva and could infect susceptible subjects via direct contact or aerosols [78].
- Human T-lymphotropic virus is involved in adult T cell leukaemia and spastic paraparesis. This virus can be transmitted through blood [79,80,81,82] and, in a dental setting, it can infect via sharp instruments injuries [10].
- Hepatitis B virus (HBV) causes acute hepatitis and it is an important risk-agent for the health care staff [83,84]. The possible ways of transmission are sexual intercourse, through blood, contaminated material injuries, and perinatal way [85,86,87,88]. So, all operators of the dental team should be vaccinated [10,89].
- Hepatitis C virus (HCV) causes “non-A” and “non-B” hepatitis and it is transmitted like HBV [85,86,87,90]. The primary infection is often asymptomatic and the most of infected subjects become carriers of the virus with risk of development of chronic liver disease that could evolve in hepatocellular carcinoma [10].
- Human immunodeficiency virus (HIV) infects the immune system of susceptible subjects, T-helper cells particularly. It can be transmitted like HBV (sexual intercourse, blood borne and perinatal ways) [91,92]. Moreover, this infection have oral manifestations that can help in diagnosis: e.g., oral candidiasis, oral hairy leukoplakia, oral necrotising ulcerative gingivitis and oral Kaposi’s sarcoma [10,93,94,95,96].
- Cytomegalovirus (CMV) is part of the herpes virus family and can cause diseases with several manifestations [97].
- Mumps virus is part of the Paramyxoviridae group. This pathogen often affects the parotid glands, and the consequently characteristic symptom is swelling of these salivary glands [98]. Moreover, this virus can cause inflammation of the ovaries, testis, pancreas or meninges with several complications. After the introduction of the vaccine against measles, mumps, and rubella (MMR), mumps incidence has decreased, even if several mumps cases have recently been reported [99].

##### Bacterial infections

- *Mycobacterium Tuberculosis* causes tuberculosis and is a bacterium transmitted by inhalation, ingestion and inoculation. The main symptoms are cervical lymphadenitis and lung infections. In order to prevent infection, the dental team should be adequately vaccinated and wear PPE [100,101,102,103,104,105]. This bacterium is resistant to chemicals and, for this reason, sterilization and disinfection protocols must be rigorously performed [10].
- *Legionella* spp. is a gram-negative bacterium that causes Legionellosis and generally it resides in water tanks. Legionellosis occurs with pneumonia, sometimes lethal in older people. Since this pathogen lives in water, it can be easily transmitted during dental procedures through aerosols from incorrectly disinfected water circuits [106,107]. In fact, water circuits that remain unused for long periods of time should be checked regularly to prevent Legionella bacteria from residing [106,107].
- *Treponema Pallidum* causes syphilis and dental team must wear gloves in order to adequate protect themselves [10,108].
- Meningococcal spp. are gram-negative bacteria. They are located on the nasopharyngeal mucosa and their presence is generally asymptomatic. The bacterium is easily transmitted, especially during adolescence, when people get together. As already mentioned, colonization of the nasopharynx is common, and while the resulting disease is rare, at times, it can cause death or permanent disability [109,110].
- *Staphylococcus Aureus* is an important agent involved in nosocomial infections. This bacterium causes a wide range of diseases that can be mild or life-threatening (e.g., bacteraemia, pneumonia, and surgical site infection [111]). In addition, *S. Aureus* can easily have antimicrobial resistance. This bacterium principally resides on the epithelium of the anterior nares in human beings [112].
- Group A streptococcus (GAS) is a gram-positive, beta-haemolytic bacterium. This pathogen is responsible for several diseases in human beings, such as acute pharyngitis, impetigo and cellulitis. It can also cause serious invasive diseases such as necrotizing fasciitis and toxic shock syndrome (TSS) [113,114,115]. The bacterium mainly resides in human nose, throat and on skin and it is often transmitted without symptoms [116,117,118]. Obviously, asymptomatic subjects are less contagious than the symptomatic carriers of this bacterium. GAS is transmitted through respiratory droplets spread in the air, for example during coughing, sneezing and nasal secretions [117,118]. In addition, this bacterium can spread through close interpersonal contact during a kiss, using the same dishes and sharing the same cigarette.
- *Streptococci Mutans* mainly colonize dental surfaces after tooth eruption and is associated to the development of caries [119]. This bacterium may be transmitted horizontally between children during the initial phases of the *S. Mutans* colonization in nursery environments [120]. There is scientific evidence of vertical transmission of *S. mutans* from mother to child [121].
- Some periodontal bacteria (e.g., *A. actinomycetemcomitans*, *P. gingivalis*) are considered person-to-person transmitted, but it is still unclear if transmission is governed only by domestic pathways, without definitive implications for the dental office. Vertical transmission of *A. actinomycetemcomitans* is between 30% and 60%, while that of *P. gingivalis* is rarely observed. Horizontal transmission ranges from 14% to 60% for *A. actinomycetemcomitans* and between 30% and 75% for *P. gingivalis* [122]. Certainly, by understanding the spread mechanisms of these bacteria, it would also be possible to prevent a number of systemic diseases [123].

### 3.2. Personal Protective Equipment (PPE) 

The dental team must adapt several precautions to avoid these infections; an adequate training and information of the personnel is mandatory in order to control infections in the dental office. The individual protection methods include a series of enforcement with the aim to reduce the risks of contamination, unfortunately without being able to eliminate them. The basic principle of infection control is to approach to each patient as if he was an infected patient (by one of the main microbes listed above) and to correctly carry out the protection methods [124].

Adequate personal protective equipment (PPE) must be selected based on a risk assessment and the procedure to be performed. The precautions for infection control require wearing gloves, aprons, as well as eye and mouth protection (goggles and mask, such as medical masks and Filtering Face Piece or FPP) for each procedure involving direct contact with the patient body fluids. Whenever possible “single use” or “disposable” equipment should be used [10] (Table 2).

#### 3.2.1. Mask/Respirators

If the necessary precautions are not taken, it is inevitable that operators can become infected through contact of the mucous membranes with blood, saliva, and aerosols from a potentially infective patient [10]. In healthcare setting, masks are used in order to:

1. protect personnel from contact with patient infectious material;
2. protect patients from infectious agents carried by healthcare workers;
3. limit the potential spread of infectious respiratory aerosol between patients [19].

Masks can be worn with goggles in order to protect mouth, nose and eyes, or with a face shield to provide more complete face protection. We must distinguish masks from particle respirators that are used to prevent inhalation of small particles which may contain infectious agents transmitted through the respiratory tract. The mouth, nose, and eyes are sensitive portals to the entry of infective pathogens, such as skin cuts. 

Medical masks:

- could be flat or pleated (some are like cups) and fixed to the head with straps or elastic bands;
- does not offer complete protection against small particle aerosols (droplet nuclei) and should not be used during contact with patients with diseases caused by airborne pathogens;
- they are not designed to isolate the face and therefore cannot prevent inhalation by the health personnel wearing them;
- they must be replaced if wet or dirty.

There are no standards that evaluate the efficiency of the medical mask filter. AORN (Association of peri-Operative Registered Nurses) recommends that medical (surgical) masks filter at least 0.3 µ particles or have a bacterial filtration efficiency of 90%–95% [126]. 

Surgical masks (SM) are used to prevent that large particles (such as droplets, sprays or splashes), containing pathogens, could reach nose and mouth [127]. Although their purpose is to protect patients from healthcare professionals (and healthcare team from patients) by minimizing exposure to saliva and respiratory secretions, they do not create a seal against the skin of the face and therefore are not indicated to protect people from airborne infectious diseases.

Masks are available in several shapes (modeled and unprinted), dimensions, filtration efficiency and attachment method (ribbons, elastic through the ear). Masks are disposable and must be changed for each patient.

Instead, during the treatment of patients with respiratory infections, particulate respiratory masks must be worn.

Particulate respirators (with filtering percentage) in use in various countries include:

- P2 (94%) and P3 (99.95%) in Australia and New Zeland
- II (95%) and I (99%) in China
- CE-certified FFP class 1 (FFP1) (80%), class 2 (FFP2) (95%), or class 3 (FFP3) (99.7%) in European Union
- 2nd class (95%) and 3rd class (99.9%) in Japan
- 1st class (94%) and special respirators (99.95%) in Republic of Korea
- National Institute for Occupational Safety and Health (NIOSH)-certified N95 (95%), N99 (99%) and N100 (99.7%) in United States [126].

FFP2 European respirators are comparable to N95, and they are indicated for prevention of

infectious airborne diseases. However, FFP3 respirators offer the highest level of protection against infectious agents and are the only FFP class accepted by the Health and Safety Executive (HSE) as regards the protection in the healthcare environment in the United Kingdom [126].

The powered air purifying respirator is also considered a standard part of PPE in certain situations, including aerosol generation procedures in high risk environments.

European legislation:

Particulate respirator masks are subject to compliance with directive 89/686/EEC about personal protective equipment (the directive on medical devices 93/42/EEC applies instead to surgical masks).

According to the British standard EN 149:2001 (modified in 2009) they are classified into three categories, FFP1/FFP2/FFP3, based on their level of protection and their effectiveness.

On each particulate respirator mask must be present:

- Name of the manufacturer
- Reference standard nuber (e.g., EN 149:2009)
- class (e.g., FFP1, FFP2 or FFP3)
- CE mark
- Possible reuse (NR or R)

In the event of a pandemic infection, any aerosol generation procedure on infected patients should only be carried out with an FFP3 respirator. Non-urgent procedures should be postponed until the infection resolves.

In the US, the National Institute for Occupational Safety and Health (NIOSH) defined the following particulate filter categories in 2011, in Title 42 Code of Federal Regulations, section 84 (Table 3).

There are several models of FFP2 and FFP3 respirators, both with valves and without valves. However, this is not a filter but a valve that regulates the flow of air at the outlet and therefore makes it easier to exhale. Therefore, these masks are designed to be able to filter very well the air that comes in the mouth, nose, and lungs of those who wear them. Instead, these masks are not designed specifically to prevent the wearer from infecting someone else with their own breathing.

In practice, if a mask has a valve, it can let out particles, even if it manages to block almost all the inlet ones. And therefore, a healthy person can use it effectively so as not to get infected. For a sick person or one who could be contagious, however, using it could infect others by letting germs pass from their breath outwards. It is important to say that there is no specific test that has been done to verify the possibility that the virus spreads from an infected person passing through a mask equipped with a valve [128]. 

Surgical masks, on the other hand, are similar in both directions. They have been designed to prevent healthcare workers and surgeons in particular from infecting their own breath with patients, who may have open wounds on the operating table, but also work to protect the healthcare staff themselves against a potentially contagious person. Their effectiveness, however, is much lower also because they do not prevent the breath from spreading and allow a lot of air to pass through and to the mouth and nose [128].

#### 3.2.2. Goggles, Face Shields

The choice of individual eye protection devices (such as goggles or face mask) varies according to the exposure circumstances, other PPE worn. and the need for personal vision [10]. In order to protect the eyes, eyeglasses and contact lenses are not considered suitable [129]. Eye protection must be effective but at the same time comfortable and allow sufficient peripheral vision.

There are different measures that improve the comfort of the glasses, for example anti-fog coating, different sizes, the possibility of wearing them on prescription glasses. Although they provide adequate eye protection, glasses do not protect from splash or spray the other parts of the face.

Disposable or sterilizable face shields can be used in alternative to glasses. Face shield protects the other areas of the face besides the eyes (glasses only protect the eyes). The face shields that extend from the chin to the forehead offer better protection of the face and eyes from spray and splashes [83].

The removal of a facemask, goggles, and mask can be safely performed after removing dirty gloves and after performing hand hygiene.

#### 3.2.3. Gowns or Coveralls

Gowns and coveralls are additional personal protective equipment in the health sector [83]. Operator hygiene, including wearing appropriate clothing and PPE, has a dual purpose: on the one hand, to defend the operator himself in an environment where the infectious risk is high, and on the other hand to prevent the operator from becoming responsible transmission of infections.

To increase the protective function of the uniform or to carry out those procedures in which high contamination is expected, additional disposable clothing can be worn [83]. These clothes can be PPE certified for biological risk and for this recognition must comply with the requirements of the technical standards, namely European standards are EN 14126 and ISO 16604 (DPI) and EN 24920 (DM). The material constituent is mainly TNT (texture not texture), which is suitable for “disposable” use in this specific area. To offer greater protection of the part front of the body, the most exposed to risk, it is required that such lab coats have standard features within the heterogeneity of the models, for example: back closure, covered or heat-sealed seams, long sleeves with cuffs tight and high collar. Obviously, for these devices, comfort and practicality are also required, so the operator must be able to move freely and perceive good perspiration [83].

Different types of gowns and overalls are available with varying levels of protection. The level of protection depends on various factors including the type of tissue, the shape and size of microorganisms, the characteristics of the conveyor, and various external factors [130]. 

In high-risk environments, it is recommended to use waterproof and fluid-resistant gowns or overalls.

During minor oral surgery, surgical gowns must be worn with tight cuffs that must be inserted under the gloves. Fabric work uniforms must be washed daily on a hot 60 ° C cycle. Fabric uniforms are not considered PPE since the material they are made of is absorbent and therefore offer little protection against infectious pathogens.

#### 3.2.4. Gloves

During all dental procedures, it is impossible to avoid contact of the hands with blood and saliva [10]. That is why all operators must wear protective gloves before performing any type of procedure on patients [10]. Gloves must be changed with each patient and at every contact with contaminated surfaces to prevent cross-infection [10]. Not only the dentist, but also other dental team members must wear gloves during dental procedures [10,131].

Gloves used in dental clinic can be distinguished basically in two categories: those for purely use clinical and those for instrumentation reordering procedures and of the operational area. When cleaning dental appliances and instruments, more durable gloves should be worn than normal non-sterile gloves to prevent injury [10].

Regarding clinical gloves, a clear distinction must be made between them procedures that require invasive action on the patient, or however at clear biological risk, and the procedures that do not require them, or in any case present a negligible biological risk for the operator.

The two types of gloves resulting from this distinction are found in the words “inspection gloves” and “surgical gloves” one commonly used nomenclature [83].

Both disposable products, from a macroscopic point of view usually have some obvious differences:

- Surgical gloves in general always distinguish the right side from the left, they are long enough to be worn over the cuffs of the gowns and always packaged in sterile pairs,
- The inspection glove is usually an ambidextrous device, shorter and thinner than the previous one and rarely sterile [132].

In general, clinical gloves are made of latex, nitrile or vinyl. Latex and nitrile have proven to be more resistant than and therefore are generally preferred. Gloves contain powder to make them easier to wear, but which can cause skin irritation [10]. Powder-free gloves exist on the market and they should be used when such reactions occur [10]. Some people may experience allergies and contact dermatitis due to latex [10]. Latex-free gloves for allergy sufferers are also available [10].

Also, the weather of use is an absolutely relevant parameter in terms of protection. The use of the glove, especially if in latex, involves development not perceived of microperforations which become particularly significant from a numerical point of view after 60 min and which induce an increase in biological risk [133]. The simultaneous use of two pairs of gloves considerably reduces the passage of blood through microperforations [134]. There are no significant reductions in manual skills and the sensitivity of the operator wearing the double glove [132].

It was confirmed that the formation of microperforations can be also induced by washing gloves with soap, chlorhexidine, or alcohol. Moreover, particular attention should be paid also while waiting for the total drying of the alcoholic substances applied on the hands, which has also proven to be potentially harmful to the integrity of the device, before wearing gloves [132].

Other personal protective equipment include the disposable cap (headgear) and shoe covers.

A disposable cap device is recommended for clear hygienic reasons, such as containment operator contamination and prevention of dispersion of dandruff in the environment, and even more generic protective functions for the worker, such as: interlocking with subsequent tearing of hair and possibly scalp from a part of moving and/or rotating organs, the burning of the hair due to flames or incandescent bodies, and hair fouling due to various agents, including powders and drops of blood-salivary material [83].

### 3.3. Personal Hygiene

Dentist personal hygiene is an absolute necessity for infection prevention [23]. The image that the doctor presents of himself and his study is related to the trust that the patient will show towards the doctor and the treatment itself, in an era in which there is increasing information and awareness of the risk. Specific notes of hygiene include:

- hair, if a doctor hair can touch the patient or dental equipment, should be attached to the back of the head or a surgical cap should be worn [23];
- facial hair should be covered with a mask or shield [23];
- jewels should be removed from the hands, arms, or facial area during the patient treatment [23];
- nails should be kept clean and short to prevent the perforation of the gloves and the accumulation of debris [23];
- full forearm and hand washing are mandatory before and after treatment [23].

#### Hand Hygiene

It is very important to maintain an excellent level of hand hygiene in protection techniques that affects all members of the dental team [10]. “Hand hygiene” includes several procedures that remove or kill microorganism on the hands [83]:

- during handwashing, water and soap should be used in order to generate lather that is distributed on all surface of the hands and after rinsed off;
- hand antisepsis, to physically remove microorganisms by antimicrobial soap or to kill microorganisms with an alcohol-based hand rub;
- surgical hand rub procedure that kills transient organisms and reduces resident flora for the duration of a surgical procedure with antimicrobial soap or an alcohol-based hand rub [135].

There are different types of soap:

- plain soap, that have no antimicrobial properties and works physically removing dirt ad microorganism;
- alcohol-based hand rub, used without water, kills microorganism but does not remove soil or organic material physically;
  antimicrobial soap kills microorganism and removes physically soil and organic material [135].

In 1975 and in 1985, the CDC published a guideline on how to wash the hands, stating that the hands should be washed with antimicrobial soaps before and after procedures performed on patients [10].

The use of gloves is not an alternative to hand washing [10].

Hand washing is different if it is a routine procedure or a surgical procedure: in the first case, normal or antibacterial soaps are sufficient [89].

Alcohol-containing agents are preferable [10]. Cold water must be of choice when washing hands because the repeatedly use of hot water can cause dermatitis [10].

It is recommended to wash hands using liquid soap for a minimum duration of 60 s. It is very important to reduce the number of microorganisms before each surgical procedure; that is why applying antibacterial soaps and acts a detailed cleaning followed by liquids containing alcohol is recommended [10]. Despite the fact that the antibacterial effects of alcohol containing cleansers arise quickly, such antiseptics including compounds of triclosan, quaternary ammonium, chlorhexidine, and octenidine must be included [10].

Before surgical hand washing, rings, watches, and other accessories must be taken off and no nail polishes or other artificial must be present [11,89].

The use of disposable paper towels is preferable for drying hands.

After every procedure and after taking off the gloves, it is highly recommended to wash hands once again with regular soaps.

If soap and water are not readily available, it can be used an alcohol-based hand sanitizer that contains at least 60% alcohol [10]. 

### 3.4. Safety of Tools

#### 3.4.1. Sharp Safety

Recommendations for sharps safety in Dental Settings by CDC [136]:

- must consider all sharp objects contaminated with the patient blood and saliva as potentially infectious;
- do not hood the used needles in order to avoid an accidental injection [83];
- put all used sharp objects in suitable puncture resistant bins [83].

#### 3.4.2. Instrument Sterilization 

It is necessary to clean all instruments with detergent and water before sterilization [10]. During washing, it is advisable to avoid splashes of water a wear gloves and face protection. The instruments that penetrate the tissues must be sterilized in an autoclave [83]. It is advisable to heat sterilize items that touch the mucosa or to at least disinfect them, for example, with the immersion in a 2% glutaraldehyde solution in a closed bid, naturally following the instructions of the producer [83]. Anything that cannot be autoclaved must be disinfected. The handpieces should be able to drain the water for two minutes at the start of the day. Not autoclavable handpieces can be disinfected using viricidal agent. After sterilization, all instruments must be kept safely in order to avoid recontamination for a maximum of 30 days, 60 days if closed in double bags [83].

Sterilization completely kills all vital agents and spores too. The classic sterilization procedure expects the use autoclave, with cycles at 121 °C for 15–30 min, or at 134 °C for 3–4 min [23,83]. It is necessary to thoroughly wash and dry all items before sterilizing them as dirt and water can interfere with sterilization [83].

Steam sterilization cannot be used for all facilities and a possible alternative can be the use of chemical sterilization using ethylene oxide gas, formaldehyde gas, hydrogen peroxide gas, liquid peracetic acid, or ozone [83]. The disinfection processes do not destroy the bacterial load, rather reducing it to acceptable levels. Commonly used disinfectants are described below (Table 4).

The action of cleaning and disinfection can be manual or automatized. For example, it is possible to use ultrasonic baths in order to clean complex, articulated, or notched stainless-steel instruments such as cutters. The washer-disinfectors provide a high temperature passage (generally 90 °C for one minute), which drastically reduces the microbial contamination of the items. The final rinse must be carried out with high quality water (Table 5).

### 3.5. Operative Room Protection

#### 3.5.1. Surface Asepsis/Disinfection

It is necessary to have always a perfect protection of operative room with disinfected surfaces [10].

There are two ways to make a surface aseptic [23]:

- Clean and disinfect contaminated surfaces [23] and
- Prevent surfaces from being contaminated by using surface covers [23].

A combination of both can also be used [23]. 

The following chemicals are suitable for surface and equipment asepsis:

- Chlorine, e.g., sodium hypochlorite
- Phenolic compounds
- Water-based, Water with ortho-phenylphenol, tertiary amylphenol, or O-benzyl–p-chlorophenol
- Alcohol-based ethyl or isopropyl alcohol with ortho-phenylphenol or tertiary amylphenol
- Iodophor–butoxy polypropoxy polyethoxy ethanol iodine complex [23].

In the literature there are still little information on 2019-nCoV. Similar genetic features between 2019-nCoV and SARS-CoV indicate that COVID-19 could be susceptible to disinfectants such as 0.1% sodium hypochlorite, 0.5% hydrogen peroxide, 62%–71% ethanol, and phenolic and quaternary ammonium compounds [4]. It is important to pay attention to the duration of use, dilution rate, and especially the expiration time following the preparation of the solution [4]. A recent paper pointed out that surface disinfection could be performed with 0.1% sodium hypochlorite or 62%–71% ethanol for one minute in order to eliminate SAS-CoV-2 [139].

After each treatment, work surfaces should be adequately cleaned and decontaminated with ethyl alcohol (70%). If blood or pus is visible on a surface, it is necessary to clean and disinfect that surface with sodium hypochlorite (0.5%). It is necessary to wear protective gloves and care taken to minimize direct skin, mucosal or eye contact with these disinfectants.

In addition to disinfection with chemicals, a Ultraviolet-C (UV-C) irradiation lamp can be used [140]. The UV light system for disinfection has several advantages, including: does not require room ventilation, does not leave residues after use and have a wide action spectrum in a very short time [140]. The UV-C lamp must be activated only when the room is empty, without staff and without patient. In the literature, there are no cases of damage to the materials present in the room; despite this, the acrylic material can be degraded if subjected to repeated exposure to UV-C light and for this reason it is recommended to cover it during disinfection with UV-C [141]. Ultraviolet light has a wavelength between 10 and 400 nm, while ultraviolet-C (UV-C) light has a wavelength between 100 and 280 nm, and the greatest germicidal power is obtained with a wavelength of 265 nm [142]. The germicidal effect of UV-C light causes cell damage thus blocking cell replication [141]. In descending order of inactivation by UV-C light, there are bacteria, viruses, fungi, and spores [143]. UV-C rays can be generated by low pressure mercury lamps and pulsed xenon lamps which emit high intensity pulsed light with a higher germicidal action [141]. UV-C rays are equipped with high energy which decreases exponentially with the increase of distance from the light source: objects or surfaces closer to the UV-C source will have a greater exposure and therefore will have to be disinfected for less time than distant objects [142].

Depending on the nature of the object affected by UV-C light, it can block the light rays or allow itself to be passed through allowing the irradiation of the objects placed behind it. For example, the organic material completely absorbs the UV-C light and blocks its diffusion. For this reason, the surfaces must be manually cleaned to remove the organic substances before decontamination with ultraviolet light [142].

The extent of inactivation of the microorganisms is directly proportional to the UV-C dose received and this, in turn, is the result of the intensity and duration of exposure [142]. Therefore, according to the data in the literature, the use of UV-C rays for disinfection has proven effective in reducing the overall bacterial count and significantly more effective than just manual disinfection on surfaces [141].

In addition, to encourage the exchange of air, it is recommended to ventilate the rooms between one patient and another. If it is not possible to allow the exchange of natural air (at least 20–30 min), forced ventilation systems with High Efficiency Particulate Air (HEPA) filters must be used, paying attention to the periodic replacement of the filters.

Recommendations for environmental infection prevention and control in dental settings [136]:

- establish a protocol for cleaning and disinfection of surfaces and environments of which health personnel must be informed;
- cover with disposable films all the surfaces that are touched during the procedures (for example switches, IT equipment) and change these protections between each patient;
- surfaces that are not protected by a barrier should be cleaned and disinfected with a disinfectant after each patient;
- use a medium level disinfectant (i.e., tuberculocidal indication) if a surface is visibly contaminated with blood;
- for each disinfectant, follow the manufacturer’s instructions (e.g., quantity, dilution, contact time, safe use, disposal) [136] (Table 6).

#### 3.5.2. Dental Unit Waterlines (DUWLs) 

If proper maintenance is not carried out, microbial pathogens (e.g., *Pseudomonas* or *Legionella* spp.) can multiply in DUWLs. These organisms grow in the biofilm on the internal surfaces of the tubes, where they cannot be attacked with chemicals. To prevent the formation of this biofilm, the systems should be drained at the end of each day [144].

In Dental Unit Water Lines (DUWL), water must flow and they must be washed regularly: it is recommended to rinse for two minutes at the beginning and end of each day and for 20–30 s between patients [144]. Different agents for disinfection of DUWL are available. All handpieces and ultrasonic meters must be equipped with backstop valves and must undergo periodic maintenance and inspection. The filters used in the DUWL must be checked periodically or, if they are disposable, they must be changed daily.

Recommendations for dental unit water quality in Dental Settings:

- use water compliant with Environmental Protection Agency (EPA) standards for drinking water (i.e., ≤ 500 CFU/mL of heterotrophic water bacteria),
- follow the recommendations for water quality monitoring given by the manufacturer of the unit or waterline treatment product,
- use sterile water or sterile saline for the irrigation during surgical procedures [136].

#### 3.5.3. Waste Management

Any waste containing human or animal tissue, blood or other body fluids, drugs, swabs, dressings or other infective material is defined as “clinical waste” and it must be separated from non-clinical waste [144]. Used disposable syringes, needles, or other pointed instruments must be disposed of in a special rigid container, in order to avoid injury to operators and operators in charge of waste disposal. The waste must be kept in a dedicated area before it is collected, away from public access, and excessive accumulation of waste must be avoided [4,144].

### 3.6. Other Precautions

The whole dental team must be vaccinated against hepatitis B in order to increase personal protection [83]. Individuals who have already been vaccinated should monitor their levels of immunity against HBV over time and make booster shots [145]. All dental health care professionals should also receive the following other vaccinations: flu, mumps (live-virus), measles (live-virus), rubella (live-virus), and varicella-zoster (live-virus) [10]. In addition, the rubella vaccine is strongly recommended especially for women who have pregnancy uncertainty [131]. The influenza vaccine is very useful for dental health professionals as they are at risk for respiratory droplets infections by working in close proximity to the patients [10]. When the COVID-19 vaccine is ready, healthcare professionals should take it. As additional infection prevention and health care worker measures, rapid tests can be used in dental practices to diagnose COVID-19 before each treatment. This is because, as mentioned above, a patient without symptoms is not necessarily a healthy patient.

## 4. From the Literature to a Novel Operative Algorithm

From all these data, it is evident that the dentist and his team need to use rigid and precise operating protocols in order to avoid infectious contagion [23]. Several authors proposed some right procedures in the operative dentistry [2,3,4,10,23,83,139,146,147,148,149]. 

For this reason, we reassume them in a precise operative protocol organized for all the patients and characterized by some defined steps:

1. Prevention of infections must be a priority in any healthcare setting and therefore also in any dental clinic. To do this, staff training and information, adequate management of resources, and use of well-defined operating protocols is necessary.
2. Adequate management of the protection for operators (and therefore also for patients) begins with the roles of the secretariat. In order to better organize the workflow, the secretariat must provide a telephone triage. It would be advisable to phone each patient to make sure he is healthy on the day of the appointment. Patients with acute symptoms of any infectious disease should be referred at the time of symptom resolution. The medical history of patients may not reveal asymptomatic infectious disease of which they are affected. This means the operator must adopt the same infection control rules for all patients, as if they were all infective. In addition, the secretariat must organize appointments in order to avoid crowding in the waiting room. It would be advisable for the patient to present himself alone, without companions (only minors, the elderly and patients with psycho-physical conditions can be accompanied). In some urgent and non-deferrable cases, it is necessary to treat the patient despite being in the acute phase of infection with any virus. Examples of urgent treatments are: pulpitis, tooth fracture, and avulsion [2]. In these cases, the operator must implement the maximum individual protection measures.
3. In the waiting room all material (e.g., magazines, newspapers, information posters) that can represent a source of contamination must be eliminated so that the room is easy to disinfect.
4. Patients are requested to go to the appointment without any superfluous objects. At the entrance of the dental structure, the patient must wear shoe covers, disinfect the hands with hydroalcoholic solution according to the following indications, affix any jacket on a special hanger and disinfect the hands again with hydroalcoholic solution. If there are several patients in the waiting room, they must be at least two meters away from each other. The correct hand disinfection procedure with hydroalcoholic solution is as follows:
  a) Apply a squirt of sanitizer in the palm of hand,
  b) Rub hands palm against each other,
  c) Rub the back of each hands with the palm of the other hand,
  d) Rub palms together with your finger interlaced,
  e) Rub the back of fingers with the opposite palms,
  f) Rotate thumbs in the other hand,
  g) Do a circle on palm with finger clasped,
  h) Once dry, hands are safe.

The same procedure is performed for washing hands with soap and water.

5. The operators must be adequately dressed in the correct PPE. Healthcare professionals will need to remove any jewel before starting dressing procedures. All the necessary PPE must already be positioned clearly visible and intact, in a room that will be distinct from the one where the undressing phase will take place. In both areas, hydroalcoholic solution and/or items necessary for washing hands with soap and water should be available. In the dressing room there must be trays for the collection and subsequent disinfection of the non-disposable PPE and special containers for the collection of waste where to dispose of the disposable PPE. A dressing and undressing procedure is described below, imagining that the dentist has to operate under a high risk of infection. Dressing and undressing procedures must be particularly considered.

Dressing Procedure:

a) eliminate jewels and personal items from the pockets of the uniform;
b) long hair must be tied and inserted into a cap not mandatory for single use (no tufts of hair must come out of the cap);
c) wear shoe covers;
d) perform social hand washing or disinfection with antiseptic gel;
e) wear the first pair of gloves of the right size;
f) wear the water repellent gown by tying it on the back without double knots (first the upper part and then the lower part, the latter must be tied on the front) being careful not to leave parts of the uniform exposed;
g) wear the mask (FFP2-FFP3) which must adhere well to both the nose and the mind;
h) put on the disposable water-repellent cap and be tied under the chin, the excess ribbons must be inserted inside the gown;
i) wear glasses/protective screen;
j) wear a second pair of gloves for direct patient assistance. These gloves must cover the cuffs of the disposable gown.

Undressing Procedure:

a) remove the second pair of (dirty) gloves being careful not to contaminate the underlying gloves;
b) gloves still worn with a hydroalcoholic solution are disinfected and a new pair of gloves is worn on them;
c) the face shield is removed: if it is disposable it should be trashed, and if it is not disposable, it should be placed in a container with disinfectant;
d) the second pair of gloves is removed without contaminating the underlying gloves;
e) the gloves are rubbed with hydroalcoholic solution and a new pair of gloves is worn;
f) disposable gown removal starting from the top, then the bottom, rolling it up to touch the inside, clean;
g) throw disposable shirts and second pair of gloves;
h) the gloves are rubbed with hydroalcoholic solution and a new pair of gloves is worn;
i) remove the water-repellent cap;
j) the gloves are rubbed with hydroalcoholic solution and a new pair of gloves is worn;
k) remove mask taking it by the elastics with the head bent forward and down;
l) both the first pair and the second pair of gloves are removed;
m) hands are disinfected with hydroalcoholic solution.

6. Before entering the surgical room, the patient must be dressed in a disposable gown and headgear worn in order to avoid any contagion on clothing and hair.
7. Before dental session patient should rinse and gargle with a specific mouthwash.
  Chlorhexidine is commonly used for pre-procedural oral rinses in dental offices, but its capacity of 2019-nCoV destruction has not yet been demonstrated [4]. Instead, pre-procedural oral rinses with oxidizing such as 1% hydrogen peroxide or 0.2% povidone-iodine are recommended [4]. So, the pre-procedural use of mouthwash, especially in cases of inability to use a rubber dam, can significantly reduce the microbial load of oral cavity fluids [3]. In fact, even if oral rinses seem to “limit” the viral load, virus can spread through the complete respiratory tract and it is not scientifically possible to guarantee that this reduction is constant during the operative manoeuvre (e.g., cough, sneezing, runny nose). Then the following pre-operative procedure is recommended to the patient: a) 1% hydrogen peroxide 15" gargle followed by 30” rinse, b) do not rinse with water at the end of the rinse and continue with Chlorhexidine 0.20% 60” rinse with final gargle of 15" [146]. At the end of the procedure, the patient must be appropriately undressed, and have another oral rinse performed before washing hands and face thoroughly.
8. After every patient, carefully clean all surfaces, starting from the least contaminated to the most potentially infected, taking care not to overlook the handles of the doors and the various drawers, worktops and all the devices used during the treatment and which are not disposable or autoclavable. Cover switches, mice, computer keyboards, and anything else that may be more difficult to clean with disposable film. The worktops must be free from anything that is not strictly necessary to perform the service. An accurate disinfection of the surfaces includes a preventive cleaning of the same in order to eliminate the soil which otherwise would not allow the disinfectant to inactivate the microorganisms [29]. In the same way, if you want to use disinfectant wipes, you must use one to cleanse and after another to disinfect. As regards spray disinfectants, the percentage of dilution and the time of application vary from product to product: you must follow the instructions provided by the company. Moreover, alcohol-based disinfectants (75%), 0.5% hydrogen peroxide, 0.1% sodium hypochlorite are recommended to be left to act on the surfaces for 1 min. Disinfect the circuits of the treatment center at each patient change. Between patients, the tubing of high-volume aspirators and saliva ejectors should be regularly flushed with water and disinfectant such as 0.1% sodium hypochlorite. Always air the rooms after each patient (at least 20–30 min) or use germicidal lamps. Clean floors with bleach at least two times a day.
9. During every procedure minimize the use of an air/water syringe: dry the site with cotton rollers when possible; use suction at maximum power (it might be an idea to use autoclavable plastic suction cannulas that have a greater suction capacity than normal disposable PVC cannulas) or use two saliva ejectors; in the case of exposed carious dentine, try to remove it as manually as possible using excavators; be sure to first mount the rubber dam, disinfect the crown with pellets soaked in 75% alcohol and recommend with the second operator to position the aspirator as correctly as possible to avoid excessive spraying and/or splashing; do not use air-polishing; avoid intraoral x-rays as they stimulate salivation, coughing and/or vomiting; prefer exams like OPT (*orthopantomography*) or CBCT (*cone beam computed tomography)*. In case of extractions, it is preferable to use resorbable sutures to seal the post-extraction site. In the case of patients who are definitely positive for any infectious agent or on which there are greater possibilities of positivity highlighted by the medical history, it is necessary to plan their treatment at the end of the day. Do not touch patient card and pens with dirty gloves. It is good practice to cough or sneeze into the elbow. The operator must avoid touching his eyes, nose and mouth with dirty gloves or hands.
10. Isolation with rubber dam [4]. Isolating the oral cavity with the use of rubber dams greatly reduces (about 70%) the spread of respiratory droplets and aerosols containing saliva or blood coming from the patient and aimed to the operator area of action [4]. After positioning the dam, the operator must provide an efficient high-volume intraoral aspiration in order to prevent the spread of aerosol and spray as much as possible [148]. If rubber dams cannot be used for any reason, the operator should prefer to use manual tools such as hand scalers [4].
11. Anti-retraction handpiece [4]. During the COVID-19 pandemic, operators should avoid using dental mechanical handpieces that do not have an anti-retraction function [4]. Mechanical handpieces with the anti-retraction system have valves (anti-retraction) that are very important in order to prevent the spread and dispersion of droplets and aerosol [148,149].
12. All instruments which have been used for the treatment of a patient or which have only been touched by operators during a session and which cannot be sterilized according to standard protocols, must be disinfected (e.g., immersed in a container with phenol) [23]. This tools bagged in disinfection solution must remain in solution for about 10 min [23]. Some materials, such as polysulphide, polyvinylsiloxane, impression compound, and ZOE impressing materials, after being in the patient mouth, are rinsed with water and immersed in a 5.25% sodium hypochlorite solution for about 10 min [23]. The alginate or polyether impressions are also rinsed with water, sprayed with a 5.25% sodium hypochlorite solution and placed in a container for about 10 min [23]. Wax, resin centric relation records, and ZOE are rinsed with water and sprayed with a 5.25% sodium hypochlorite solution and placed in a plastic bag for about 10 min [23]. Provisional restorations and complete dentures removed from the patient mouth are immersed in a 5.25% sodium hypochlorite solution for 10 min [23]. Otherwise, removable partial prostheses with metal bases are treated with 2% glutaraldehyde solution and placed in a plastic bag for 10 min [23].

A novel and useful indication is that of classifying each common dental procedure according to the likelihood of a contagion by one or more infective agents (via saliva, blood, droplets or aerosol) for the team and for the patient (under the cure or the subsequent), nevertheless its type and intrinsic operative difficulty (Table 7). 

According to this paradigm, all dental procedures involving the use of the air-water syringe and/or rotating/ultrasound/piezo tools are able to produce high levels of aerosols and droplets and for this reason the dentist must consider them dangerous for himself, the dental team, and the subsequent patients. Meanwhile, procedures, even if refined (e.g., soft tissues biopsy for oral cancer suspicion) but characterized by a low/absent production of aerosol and droplets, must be considered not particularly threatening.

For all these considerations, the dental team must reconsider its operative protocols and modulate the PPE use according to level of risk of common dental procedures of generating droplets or aerosols. Table 8 presents the use of different PPEs for each common dental procedure in pre-COVID vs post-COVID era. It is definitively clear that the use of air-water syringe and/or rotating/ultrasound/piezo tools able to produce high levels of aerosols and droplets need the use of the safest PPE in order to reduce/eliminate viral or other infectious agent diffusion within the dental setting.

## 5. Conclusions

In the face of the COVID-19 pandemic, new biosafety measures are necessary to reduce contagion. Dentistry is a profession that works directly with the oral cavity and is therefore very exposed to this virus or other infectious agents. Because of this, some measures need to be taken to minimize contagion. In fact, dentists can play an important role in stopping the transmission chain, assuming correct procedures in order to reduce the viral agent diffusion, or in promoting undesirable infectious disease diffusion, if operating in adherence to adequate safety protocols. Dental-care professionals must be fully aware of 2019-nCoV and other viral agent spreading modalities, how to identify patients with active infections and, most importantly, to prioritize self and patient protection. Finally, the dental team must reconsider the overall infective risk level of every dental procedure and respect the new operative protocols that are or will be formulated by respective national official committees [150,151] in order to reduce as much as possible the risk of the contagion for the health and safety of their community.

## Figures and Tables

**Table 1:** Infectious agents and modes of transmission.

Infective agent	Modes of Transmission Direct Contact and/or Blood and/or Droplet	Detectable in Aerosols	Persistence on Inanimate Surfaces
Adenoviruses	Direct contact/Droplet		1–3 days
Coxsackievirus	Direct contact/droplet		7–10 days, up to >2 weeks
Cytomegalovirus	Direct contact with saliva/urine /droplet		few hours–7 days
Epstein-Barr Virus	Direct contact/droplet		few hours–7 days
Hepatitis B Virus	Direct contact with Blood		approx. 1 week
Hepatitis C Virus	Direct contact with blood		approx. 1 week
Herpes Simplex Virus	Direct contact/droplet		few hours–7 days
Human betaherpesvirus 6	Direct contact/droplet		
Human Immunodeficiency Virus	Direct contact		approx. 1 week
Human Rubulavirus (Mumps)	Droplet		
Human T-Lymphotropic Virus	Direct contact		
Influenza A-B Virus	Droplet (Airborne)	up to 3 h	8 h –3 days
MERS-CoV	Droplet (Airborne)	up to 1–3 h	1–3 days
Rhinovirus	Droplet		1–3 days
Rubella virus	Airborne		
SARS-CoV	Droplet (Airborne)	up to 1–3 h	1–5 days
SARS-CoV-2	Droplet (Airborne)	up to 1–3 h	up to 3 days
Varicella Zoster Virus	Airborne		
Legionella pneumophila	Small droplets of water in the air		
Mycobacterium tuberculosis	Airborne	30 min–24 h	1–4 moths
Neisseria meningitidis	Droplet		1–3 days
Staphylococcus aureus	Direct contact		7 days–7 months
Streptococcus spp.	Droplet		3 days–6.5 months
Treponema pallidum (Syphilis)	Direct contact		

**N.B.: Direct contact** occurs through skin-to-skin contact, kissing, and sexual intercourse.

**Table 2:** The types of PPE commonly used for high-risk settings are shown with each advantages and disadvantages.

TYPE OF PPE	ADVANTAGES	DISADVANTAGES
Medical mask	Easy to wear, disposable, comfortable compared withN95, N99 respirator or PAPR	Controversial adequacy against novel influenza or highly virulent droplet pathogens, not indicated when operator is in contact with highly virulent pathogens during aerosol-generating procedure
Particulate respirators (FFP2, FFP3, N95…)	Indicated for airborne pathogens, able to protect from virulent pathogens during aerosol-generating procedure, disposable	Less comfortable, facial hair and facial deformity prevent sealing mask to face
Powered air purifying respirator (PAPR)	Desired for high-risk aerosol-generating procedures, half or full face piece provides facial protection	Unwieldy, battery-operated, not disposable
Gown	Easy to put on and take off, not causing heat, disposable, more available	Have more openings than coveralls
Coverall	Covers large part of surface area	Causes heat stress unwieldy
Apron	Additional protection when using gowns or coveralls	Disinfection is needed with apron not disposable
Goggles	Easy to wear, Protection to eyes	Affect visibility with fogging, some parts of face may not be protected
Face shield	Less fogging, Easy to wear, covers larger part of face	
Gloves (double gloving)	Reduction of the risk of transmission for high virulent pathogens through glove holes, reduction of contamination risk for hands when removing gloves	Reduction tactile sensation, unwieldy removal process
Head and neck cover	Protects head, neck skin and hair	No evidence about protection in high-risk
Boots	Easy to disinfect, considered a standard equipment in high-risk procedures	Lack of information in comparison boots vs shoes with covers
Shoes with covers	Easy to wear	Not optimal when floors is wet

(modified from Honda et al., 2106) [125].

**Table 3:** Characteristics of masks according to US classification.

Oil Resistance	NIOSH Class	Filtration Percentage % Filtration of Airborne Particles
Not oil resistant (N)	N95 N99 N100	95% 99% 99.97%
Somewhat resistant to oil (R)	R95 R99 R100	95% 99% 99.97%
Strongly resistant to oil (P)	P95 P99 P100	95% 99% 99.97%

**Table 4:** commonly used chemical disinfectants.

	Concentration of the Preparate	Level of Activity on Target Agents	Other Characteristics	Recommended Uses
Alcohol	70%	Bacteria (high) Tubercle bacilli (high) Spores (low) Fungi (high) Viruses (active only on some viruses)	Volatile with fast action low penetration into organic matter Inflammable	Disinfection of clean surfaces and skin
Diguanides	Chlorhexidine - Aqueous 1:1000 Chlorhexidine - 0.5% in 70% Ethanol Chlorhexidine + Cetavlon - Aqueous 1:100, 1:30 Chlorhexidine + Cetavlon - 1:30 in 70% Ethanol	Bacteria (high for gram-positive) Tuberculosis bacilli (low) Spores (low) Fungi (high) Viruses (low)	Inactivated by organic matter, soap and anionic detergents	Disinfection of skin and mucous membrane Use opened bottle of aqueous skin disinfectant for maximum 24 h
Glutaraldehyde	2%	Bacteria (high) Tuberculosis bacilli (high) Spores (high but slow) Fungi (high) Viruses (high)	Slow penetration of organic matter Irritation of eyes, skin and respiratory mucosa Alkaline solution requires activation and has a limited useful life (14–28 days)	Disinfection of selected not autoclavable instruments Use only closed containers to reduce the escape of irritant vapours
Hypochlorites	1% (one part of 5.25% hypochlorite solution in 4 parts of water) 0.1% (one part of 5.25% hypochlorite solution in 49 parts of water)	Bacteria (high) Tuberculosis (high) Spores (high) Fungi (high) Viruses (high)	Inactivated by organic matter Corrosive on metals Diluted solutions decay rapidly and should be made up daily Addition of ammonia or acids causes release of toxic chlorine gas	instrumental disinfection for selected items

Modified from: Guidelines on Infection Control Practice in the Clinic Settings of Dept of Health. 2019. [137].

**Table 5:** instrument disinfection procedures.

ITEM	RECOMMENDED METHOD	ALTERNATIVE METHOD
Articulators	scrub with 70% ethyl alcohol	
Burs–diamond	Clean with metallic brush and detergent, autoclave	
Burs–steel tungsten-carbide	Clean with metallic brush and detergent, rinse, dry and dry heat	Clean with metallic brush and detergent, rinse, dry and immerse in 2% glutaraldehyde for 10 h, rinse
composite carriers	Wipe with 70% ethyl alcohol	
Dental mirrors	Clean with detergent and water, autoclave, store in covered pack or container	
Denture	Clean with detergent and water	
	If contaminated with blood, immerse in 0.1% sodium hypochlorite for 10 min and rinse	
Extraction Forceps	Clean with detergent and water, autoclave, store in covered pack or container	
Handpieces Air motor for slow speed handpieces	Flush for 30 s, clean with detergent and water, oil, autoclave	Flush for 30 s, clean with detergent and water, oil, surrounding the handpiece by a gauze pad soaked in 2% glutaraldehyde for 10 min, rinse with water
Impressions–Alginate (plastic trays)	Rinse, spray with 0.1% sodium hypochlorite, put in closed container for 10 min.	
Zinc-oxide eugenol paste	Rinse, spray with 0.1% sodium hypochlorite, put in closed container for 10 min.	
Alginate (metallic trays)	Rinse, spray with 2% glutaraldehyde, put in closed container for 10 min.	
Rubber base	Rinse, immerse in 2% glutaraldehyde for 10 min, rinse	
Instrument trays	Clean with detergent and water, autoclave	
Orthodontic bands	Clean with detergent and water, autoclave	
Orthodontic pliers	Clean with detergent and water, autoclave	
Polishing stones	Clean with detergent and water, autoclave	
Prophylactic cups and brushes	Disposable	Clean with detergent and water autoclave
Protective, plastic glasses and shields	scrub with 0.1% sodium hypochlorite	
Root canal instruments	Clean with detergent and water, autoclave, store in covered container	
Rubber dam clamps	Clean with detergent and water, autoclave	
Rubber dam forceps	Clean and autoclave	Clean, immerse in 2% glutaraldehyde for 10 min, rinse
Rubber dam punches	Clean with detergent and water	
Saliva ejectors, metallic	Clean with detergent and water, autoclave	
Stainless steel instruments	Clean with water and detergent, autoclave, store in covered pack or container	Dry heat
Suction tube adaptors	Wipe with 70% alcohol after each use. Autoclave weekly	
Surgical instruments	Clean with water and detergent, autoclave, store in covered pack or container	Dry heat
Syringe–local anaesthetic	Clean with water and detergent, autoclave, store in covered pack or container	Dry heat
Ultrasonic scaler tips and inserts	Clean with water and detergent, autoclave, store in covered pack or container	
Wax bite block, wafer	Rinse, immersion in 0.1% sodium hypochlorite for 10 min, rinse	

Table modified from DH ICCo. Guidelines on Infection Control in Dental Clinics 1993 [138].

**Table 6:** surface disinfection table.

Item	Recommended Method	Alternative Method
Attachments dental units	Clean with 2% glutaraldehyde and then rinse	Clean with 70% alcohol
Bracket tables	Clean with 70% ethyl alcohol	
	If there is blood or pus clean, disinfect with 0.5% sodium hypochlorite and rinse	
Dental chairs	Clean with detergent and water	
	If there is blood or pus clean, disinfect with 0.5% sodium hypochlorite or 2% glutaraldehyde and rinse	
Dental service unit	Wipe with detergent and water	
	If there is blood or pus clean, disinfect with 0.5% sodium hypochlorite or 2% glutaraldehyde and rinse	

Table modified from DH ICCo. Guidelines on Infection Control in Dental Clinics 1993 [138].

**Table 7:** reclassification of the risk for operative procedures in dentistry on the light of SARS-CoV-2.

Procedure	Dental Specialty	Pre-COVID	POST-COVID Risk-Level
Checks in Restraint or Post-Restraint	Orthodontics	Low	Low
Dental structure tests	Prosthodontics	Low	Low
Manual reduction of dislocation of the jaw	Gnathology	Low	Low
Mobile/fixed orthodontic appliance positioning	Orthodontics	Low	Low
Radiographic examination	Diagnosis	Low	Low
Topical periodontal therapy	Periodontics	Low	Low
Topical treatment of dental hypersensitivity and caries prophylaxis	Hygiene and prevention	Low	Low
Test of night guard/bite	Gnathology	Low	Low
Dental impression	Diagnosis	Low	Low
Prosthetic tests, positioning and adaptation (temporary/definitive, removable/fixed)	Prosthodontics	Low	Low
Biopsy	Surgery	High	Low
Bone graft (autogenous/biocompatible material) without rotating tools	Surgery	Hgh	Low
Mucogingival surgery (quadrant)	Periodontics	High	Low
Open air curettage without rotating tools (quadrant)	Periodontics	High	Low
Removal of cysts or small benign neoplasms	Surgery	High	Low
Surgical medication	Surgery	High	Low
Oral minor surgery (e.g., abscess incision, frenulectomy, frenulotomy)	Surgery	High	Low
Salivary stone removal	Surgery	High	Low
Extraction without rotating tools	Surgery	High	Low
Gingivectomy /gingivoplasty	Periodontics	High	Low
Endodontic treatment (1 root) with rubber dum (in subsequent appointment after access cavity)	Endodontics	Low	Low
Pulp hooding, pulpotomy, pulpectomy (in subsequent appointment after access cavity) with rubber dum	Endodontics	Low	Low
Bleaching	Hygiene and prevention	Low	Medium
Splinting	Hygiene and prevention	Low	Medium
Visit	Diagnosis	Low	Medium
Tartar scaling	Hygiene and prevention	Low	High
Extraction with rotating tools	Surgery	High	High
Sinus lift	Surgery	High	High
Access cavity (rotating instruments)	Endodontics	Medium	High
Implantology	Surgery	High	High
Open air curettage (quadrant) (rotating tools)	Periodontics	High	High
Resective/regenerative bone surgery (rotating tools)	Periodontics	High	High
Rhizectomy / rhizotomy (rotating tools)	Periodontics	High	High
Sealing of dental grooves	Hygiene and prevention	Low	High
Apicectomy with retrograde filling	Surgery	Medium	High
Autologous bone harvest (rotating tools)	Surgery	High	High
Abutment tooth preparation	Prosthodontics	Low	High
Odontoplasty (1 tooth)	Gnathology	Low	High
Simple / complex filling using rotating tools	Conservative	Low	High

**Table 8:** Proposal of modulation of ***personal protective equipment (PPE)*** according to level of risk or common dental procedures both in pre-COVID and post-COVID era (bold style means the introduction of the new PPE due the transition from a risk category to a higher one).

	Pre-COVID	Post-COVID
Low risk	sterilizable headgear Protective goggles Surgical mask disposable latex gloves	Disposable or sterilizable headgear Protective goggles Surgical mask Disposable or sterilizable gown Double disposable latex gloves
Medium risk	**Disposable headgear** **Disposable/sterilizable visor to remove immediately** Surgical mask Protective goggles disposable latex gloves	**Disposable headgear** **Disposable/sterilizable visor to remove immediately** **Protective respirator (FFP2)** **Disposable gown** Double disposable latex gloves
High risk	Disposable headgear Disposable/sterilizable visor to remove immediately Surgical mask **Disposable gown** disposable latex gloves	Disposable headgear Disposable/sterilizable visor to remove immediately **FPP3 / Powered air purifying respirator (PAPR)** **Disposable protective suit** Double disposable latex gloves **Cover shoes**
